# Supplementary figures and images for: Heavy metal sensitivities of gene deletion strains for ITT1 and RPS1A connect their activities to the expression of URE2, a key gene involved in metal detoxification in yeast
Source: PLoS One. 2018 Sep 19;13(9):e0198704. doi: 10.1371/journal.pone.0198704 (PMC6145592; doi:10.1371/journal.pone.0198704)

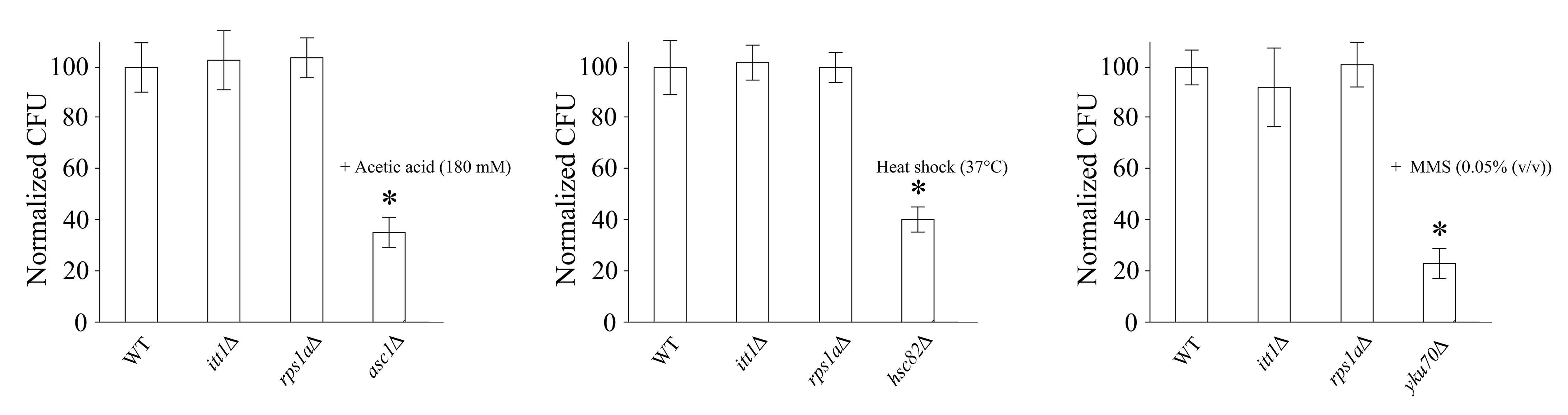

Supplement: S1 Fig — CFU counts after 3 days exposure to the experimental conditions are normalized to control condition counts. Sensitivity of itt1Δ, rps1aΔ are compared to WT phenotype in the presence of (A) acetic acid (180mM), (B) heat shock (37°C) and (C) MMS (0.05% v/v). For each experimental condition, a corresponding positive control strain is included. Each experiment was repeated at least three times. Error bars represent standard deviations. * Indicates statistically significant differences (p <0.005). (TIF) [file pone.0198704.s001.tif]

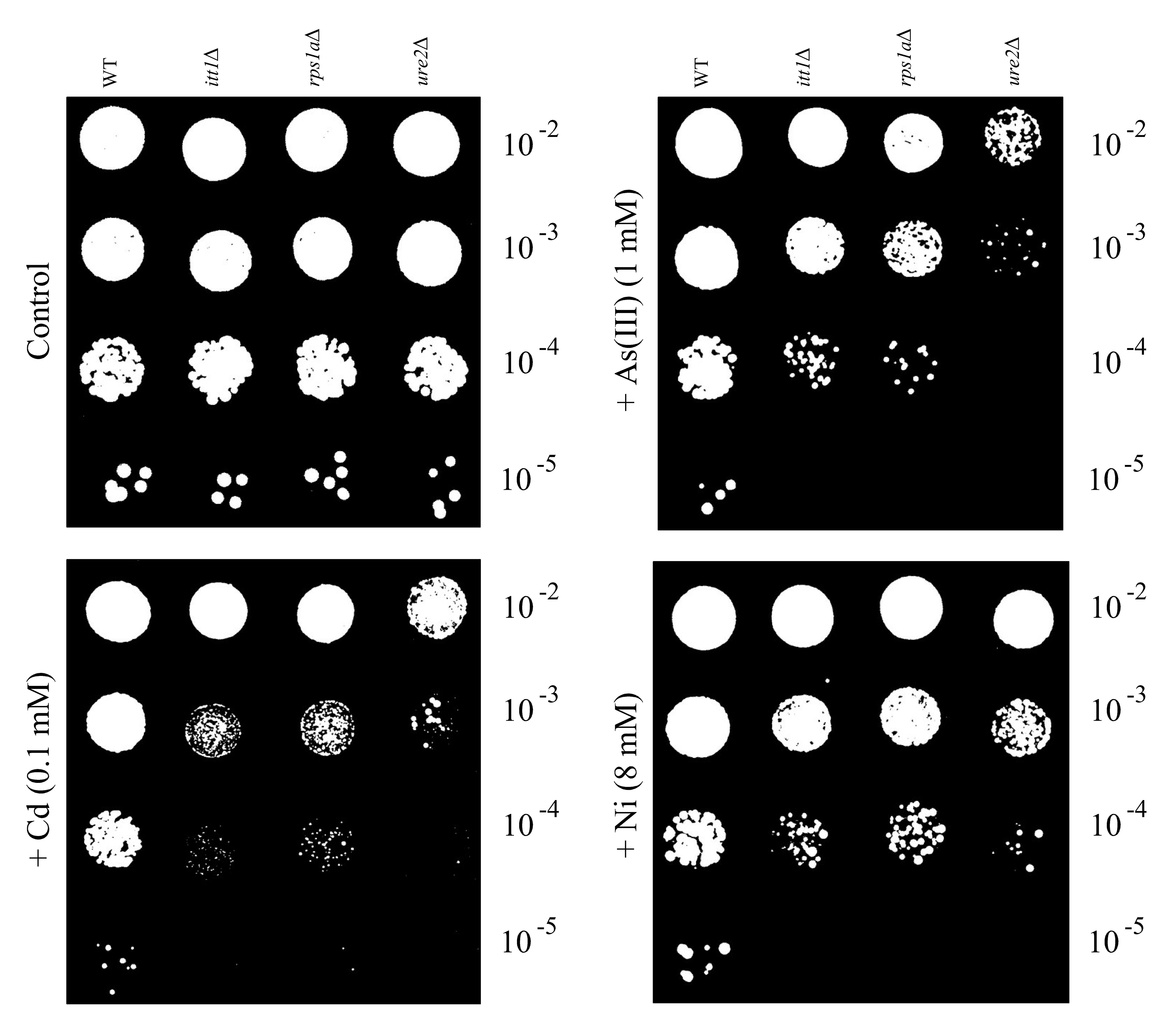

Supplement: S2 Fig — Cells were grown to saturation, serially diluted, and spotted on solid media containing As(III) (1 mM), Cd (0.1 mM), Ni (8 mM) or no drug. Plates were incubated at 30°C for 3 days. (TIF) [file pone.0198704.s002.tif]

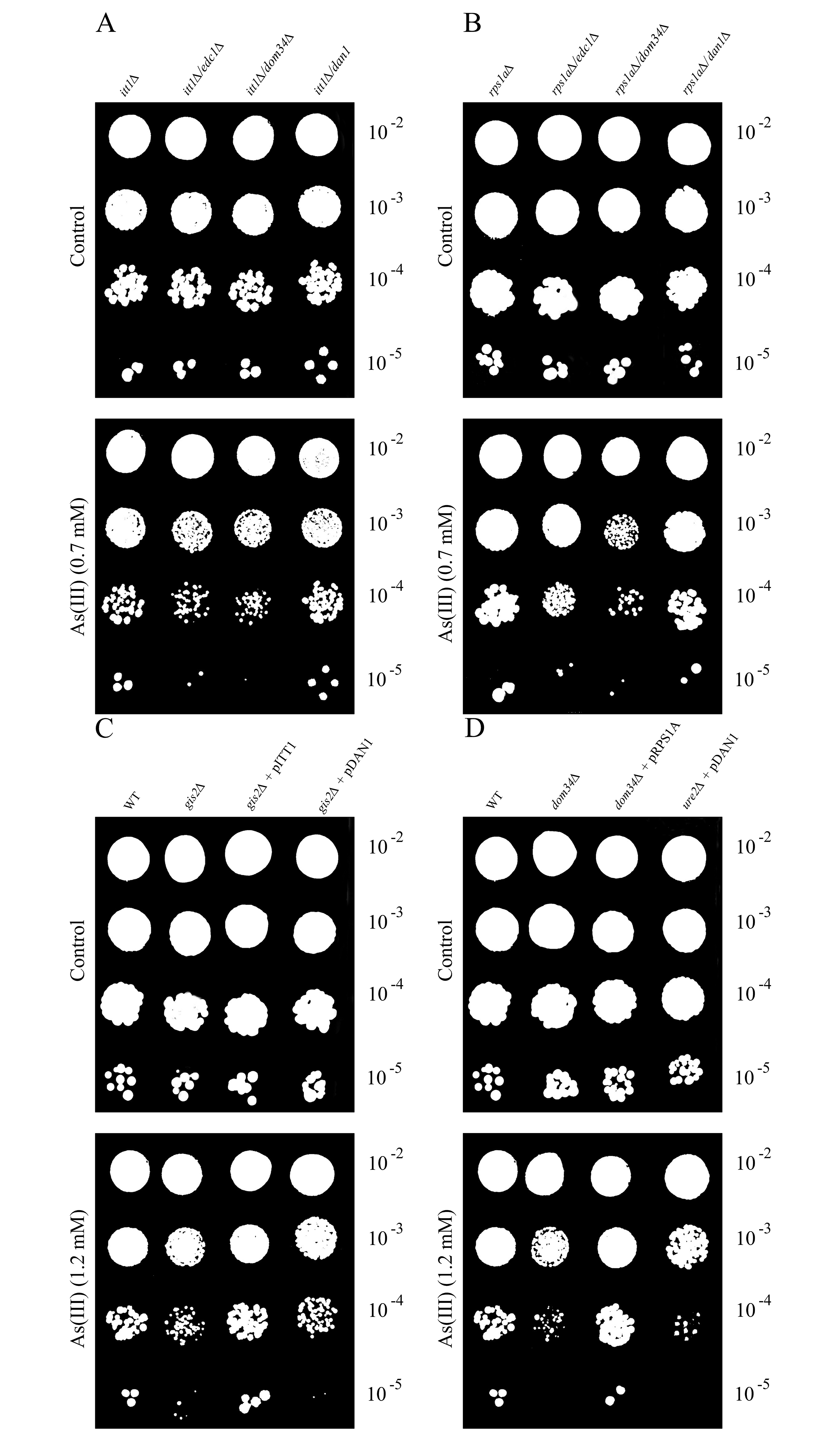

Supplement: S3 Fig — Cells were grown to saturation, serially diluted, and spotted on solid media with or without As(III). (A) and (B) EDC1 and DOM34 show conditional GI with ITT1 and RPS1A in the presence of As(III) (0.7 mM) representing conditional negative genetic interactions. DAN1 is used as a negative control. (C) and (D) Overexpression of ITT1 and RPS1A compensated the sensitivity of gene deletion strains for GIS2 and DOM34, respectively, in the presence of As(III) (1.2 mM) confirming a phenotypic suppression GI for ITT1 with GIS2 and RPS1A with DOM34. DAN1 was used as a negative control. (TIF) [file pone.0198704.s003.tif]
